# Supplementary material for: Asthma-Associated Long TSLP Inhibits the Production of IgA
Source: Int J Mol Sci. 2021 Mar 30;22(7):3592. doi: 10.3390/ijms22073592 (PMC8036615; doi:10.3390/ijms22073592)
Supplement: Supplementary file 1 [file ijms-22-03592-s001.pdf]

Supplementary Table 1. Donors used in the experiments and figures.

Exp No.	Donor	Cell Source	Figures						
			**IgA**	**IgE**	**TI IgA**	IgM, IgA, IgG1-4	FACS IgA, IgG	FACS ASC	IgA, IgG1
1	1	Cryopreserved	1B					5B	5C, 5D
2	2	Cryopreserved	1B	1C		2	3	5B	5C, 5D
2	3	Cryopreserved	1B	1C		2	3	5B	5C, 5D
3	4	Freshly isolated	1B						
3	5	Freshly isolated	1B						
3	6	Freshly isolated	1B						
3	7	Freshly isolated	1B						
4	5	Freshly isolated			1E				
4	6	Freshly isolated			1E				
4	7	Freshly isolated			1E				
5	8	Cryopreserved	1B	1C		2			
5	9	Cryopreserved	1B	1C		2			
5	10	Cryopreserved	1B	1C		2			
5	11	Cryopreserved	1B	1C		2			
6	8	Cryopreserved					3		5C, 5D
6	9	Cryopreserved					3		5C, 5D
6	10	Cryopreserved					3		5C, 5D
6	11	Cryopreserved					3		5C, 5D
